# The relationship between fear of COVID-19 and food choice motives in the Iranian population

**DOI:** 10.1371/journal.pone.0308689

**Published:** 2024-08-13

**Authors:** Seyyed-Ali Hoseinean, Bita Rahmani, Ahad Alizadeh, Maryam Javadi, Roghieh Nooripour, Atieh Razzazi, Mohammad Reza Shiri-Shahsavar

**Affiliations:** 1 Student Research Committee, Faculty of Health, Qazvin University of Medical Sciences, Qazvin, Iran; 2 Department of Nutrition, School of Heath, Qazvin University of Medical Sciences, Qazvin, Iran; 3 Medical Microbiology Research Center, Qazvin University of Medical Sciences, Qazvin, Iran; 4 Children Growth Research Center, Research Institute for Prevention of Non-Communicable Diseases, Qazvin University of Medical Sciences, Qazvin, Iran; 5 Department of Counseling, Faculty of Education and Psychology, Alzahra University, Tehran, Iran; 6 Qazvin Health Center, Qazvin University of Medical Sciences, Qazvin, Iran; 7 Metabolic Diseases Research Center, Research Institute for Prevention of Non-Communicable Diseases, Qazvin University of Medical Sciences, Qazvin, Iran; Jashore University of Science and Technology (JUST), BANGLADESH

## Abstract

**Background and aim:**

The long-term impact of COVID-19 on nutrition and community health is inevitably noticeable. These effects can change the nutritional behavior and lifestyle of survivors. Due to the COVID consequential fear and anxiety, the pandemic can alter the motivations for choosing, buying, and consuming food. The relationship between nutritional behavior and COVID-19 fear is the primary purpose of this research.

**Materials & methods:**

This cross-sectional study was conducted via online and face-to-face surveys. Accessing participants was through health centers of Qazvin, Iran, and the selected centers were sampled by cluster sampling method. The study population included 331 adults aged 18 to 65. Data were collected in three sections using valid questionnaires. The Demographic Questionnaire, FCV-19S, and FCQ were used to gather demographic information, the level of fear caused by COVID-19, and food choice motivations, respectively. The statistical analyzes were performed using R software. Analysis of variance and linear regression methods were used to determine the effect of independent variables on dependent variables (*p* = 0.05).

**Results:**

The mean score of fear of COVID-19 in the study population was 15.25 ± 5.78. Price, Mood, Natural content, Familiarity, Convenience, and Ethical concerns were significantly and positively associated with fear of COVID-19 (*p*<0.05). The only food motive significantly different than before during COVID-19 was Health, which was increased (*p* = 0.02). Sensory appeal and Health were the most important motivations for food choices before and during COVID-19. The Ethical concern was considered the least important food motivation.

**Discussion and conclusion:**

Some food motivations were associated with fear of COVID-19, possibly due to their psychological nature. The increasing importance of the Health factor and Natural content motivations can relate to the advice of experts on the importance of eating healthy food to counteract COVID-19 and indicate people’s preference for this training.

## 1 Introduction

The world is experiencing the COVID-19 pandemic as an acute respiratory syndrome. With the advent of this pandemic, many countries worldwide were forced to prevent the rapid transmission of the virus and the worsening of the situation through solid quarantine and periodical closings of public areas [1]. These closures and the announced health protocols by official organizations, health authorities, and the media have caused many slight or enormous shifts in people’s lifestyles. Moreover, This particular epidemic emerged during the global use of the internet and the growing impact of social media in disseminating information, including the spread of unconfirmed claims and unreliable fake news, often reinforced by plotted algorithms and deliberate actions. All of these exacerbate the effects of COVID-19 on public anxiety, fear, and panic [2]. Additionally, limitations and restrictive changes in routine lifestyle, regardless of the idea of getting infected with COVID-19, can affect a person’s physical and mental health in many ways. Staying home longer, reducing social relationships, increasing physical distance from family, friends, and co-workers, reducing the amount of physical activity outdoors, fear of unemployment, losing jobs, and facing financial hardships can negatively harm mental health by inducing anxiety and fear [3].

During the time dealing with this disease, such adverse matters can cause different degrees of clumsiness in the person due to the elimination of optimistic sentiments and positive emotions and the escalation in lifestyle monotony; people tend to turn to emotional overeating to get rid of monotony and fatigue of prolonged staying home, which leads to more calories consumed [2, 3]. Emotional eating is how experiencing negative emotions can lead to overeating [4].

Change in nutritional behavior due to the COVID-19 pandemic begins with the purchase of food, where people may try to buy more food in bulk [5]. This way, food storage becomes a typical behavior during the epidemy. This rational behavior occurs during the outbreak of infectious diseases to get out of the house less frequently and recede from the risk of infection. The need to store food and the fear of getting infected with COVID-19 through food may lead to other behaviors, such as baking homemade bread or buying intact, packed bread instead of buying from a traditional bakery. These behavioral changes include shopping for ready-made packaged and canned food instead of fresh, local dairy, fruits, and legumes [6–9].

Another important factor influencing food choices during the COVID-19 pandemic is food prices, conceivably due to declining incomes and unstable financial status [10]. People’s behavior can also be contradictory regarding food prices, as some prefer to eat healthy and high-nutritional foods to ensure their health. In contrast, for others, a slight change in diet and spending less money on food resulting from financial insecurities is of higher priority [11–13].

Most human behaviors are habitual, automatic connections between specific cues and responses, with a history of repetition and reward. Food choices are made daily and usually for the same purpose, thus making it a habitual response [14, 15]. Eating habits form when individuals automatically pursue goals by repeating the same answers and thoughts given to a topic that is difficult to change [16]. However, when people’s experiences change in the environment, their habits are also subjected to change as they get engaged in a new non-automated decision-making process [17]. It is hypothesized that a sudden pandemic and subsequent changes in food purchase and consumption may cause discontinuities in food choice habits [8, 18, 19].

**Fig 1:**
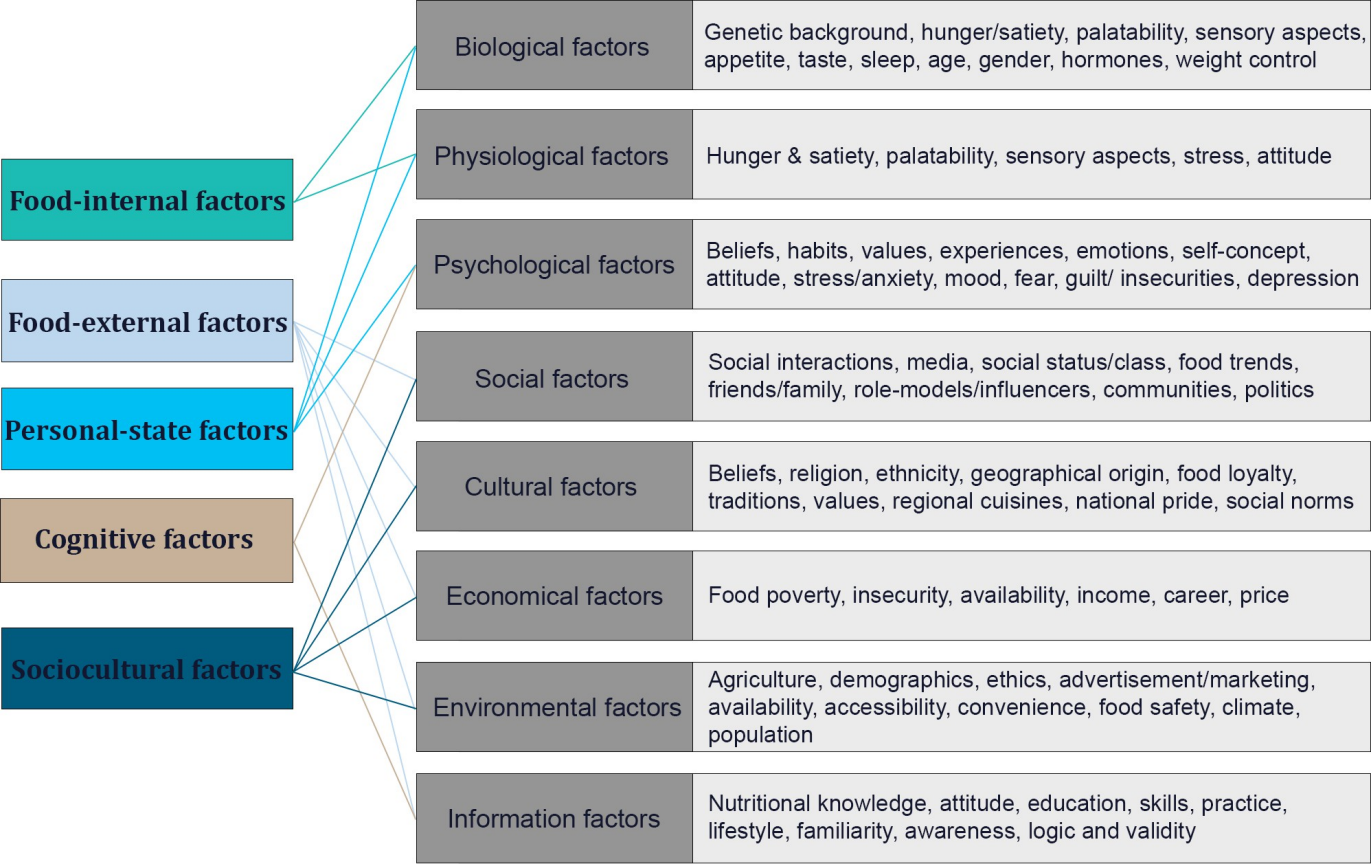
An illustration of the main factors influencing food choices and preferences.

Nutritional value is calculated by combining a set of characteristics called food motivations based on their importance to a person at the point of choice. Thus, food choice motives transitions may lead to changes in food choice decisions. Consequently, Individuals involved in a new cycle of making food choices during the COVID-19 epidemic may alter their food choices [20, 21]. Food choice motives contain various factors and considerations that influence individuals’ decisions about the foods they consume [22]. These motives extend beyond basic nutritional needs and involve a complex interplay of different categories, including food-internal, food-external, personal-state, cognitive, and sociocultural factors [22, 23] (Fig 1).

People make food choices based on a variety of motivations, such as health and nutrition, economic factors, psychological factors and taste preferences, knowledge and attitude, convenience and accessibility, cultural and social norms, and ethical and environmental concerns [10, 24, 25]. These motives collectively shape individuals’ dietary preferences, habits, and overall eating behaviors. Food choice motives include social, cultural, economic, political, and contextual factors besides the nutritional value of food [26]. Well-known motivations influencing food choices include Health, Price, Mood, Convenience, Natural content, Sensory appeal, Familiarity, Weight control, and Ethical concerns [26–30].

Accordingly, although deaths and complications from COVID-19 infection are currently more prevalent in society, other problems are occurring on a larger scale and population. The long-term impact of this disease on the community’s health after almost two years and even after the epidemic’s end is also noteworthy. Such effects may modify survivors’ food choice motivations by instilling fear of COVID-19. Nutritional changes can alter and disrupt the "farm to fork" route. The current study aimed to determine the relationship between fear caused by COVID-19 and people’s food choice motives during the COVID-19 pandemic. This study also examines the impact of disease on food choices by comparing food choice motives before and during the COVID-19 epidemic.

## 2 Materials and methods

### 2.1 Design

A cross-sectional study was conducted from October 24, 2021, to December 12, 2021− which was considered the most critical COVID-19 peak and domination of the deadly Delta variant (B.1.617.2) to that date− to investigate changes in food choice motives and their relation to fear of COVID-19 in adult residents of Qazvin, Iran. This epidemiological study was performed using online and face-to-face surveys with self-administered survey techiniques. The required information was collected using valid questionnaires. Due to the COVID-19 epidemic and its limitations and the condition to obey the health protocols, most questionnaires were provided to participants online. The study was conducted in compliance with the guidelines outlined in the Declaration of Helsinki. The study protocol was evaluated and approved by the Research Ethics Committee of Qazvin University of Medical Sciences before commencement (IR.QUMS.REC.1400.304).

### 2.2 Population and sample size

The research scope consists of 1900 people (*N* = 1900), the initial number used in the calculation. The sample size for the study was determined using Cochran’s sample size determination formula. With a 95% confidence level, a precision level of 6.16% (*E =* 0.0616), and an estimated proportion of approximately 48.98% (*p* = 0.4898), the researchers arrived at a sample size (*n*) of 331 participants. This sample size was deemed sufficient to achieve statistically significant results while considering practical constraints such as time and resources. The study population was adults aged 18 to 65 who live in Qazvin City. Based on the inclusion criteria, people willing to cooperate entered the study by completing questionnaires. The number of questions in the questionnaire was 78 [31].

### 2.3 Procedure

The place of research and access to people was through the Health Centers of Qazvin City. First, the health centers were listed according to the population number they covered, and then the selected health centers were sampled by cluster method. Each area’s number of health centers was proportional to the population covered. For online participation, the addressed link to participate in the study was sent out to people as an invitation message via the official WhatsApp channels of the health centers. Printed questionnaires were handed out to clients in COVID vaccination health centers for face-to-face participation in the study. This technique let participants fill in questionnaires while keeping social distancing and having no concerns about being infected. We provided a separate area in each of the health centers for participants who visited for COVID-19 vaccination and health purposes, to sit down in peace and the printed questionnaires were handed to them. Throughout the entire filling-in process, at least one of our researchers/personnel waited with the participants in case they sought assistance or had questions. Completing the questionnaire took approximately 10 minutes on average. The online domain limited the access of repetitive IP addresses to avoid the collection of repeated responses. Also, all participants provided their national ID numbers alongside demographic information for online and in-person participation. Their inclusion criteria variables were doublechecked using the national health and clinical check-in online systems in the health centers using participants’ national ID numbers to ensure correct demographic information. Finally, due to the optionality of cooperation, the individuals interested in entering the study completed the well-explained questionnaire with informed written consent to ensure their information confidentiality.

### 2.4 Data collection and questionnaires

The questionnaires were prepared into three sections comparing the condition of individuals before and during the COVID-19 pandemic. These sections included: Demographic information, The level of fear caused by COVID-19, and motivations to choose food.

#### 2.4.1 Personal information

Personal information was obtained using the Demographic Questionnaire, to which several questions related to the COVID-19 epidemic were added. The collected information included: age, gender, marital status, residence status, educational level, current employment status, changes in employment status, income, changes in income, and the individual’s history of COVID-19 infection.

#### 2.4.2 Fear of COVID-19

The level of fear was assessed using a 7-item FCV-19S. The range of questions is a five-point Likert scale (strongly agree = 5, agree = 4, neither agree nor disagree = 3, disagree = 2, and strongly disagree = 1). The minimum possible score for each item is one, and the maximum is five. The total score was calculated by adding each question’s score from 7 to 35. A higher score indicates a higher level of COVID-19 fear, and a lower score indicates a lower level of fear.

#### 2.4.3 Food choice motives

The Food Choice Questionnaire was used to assess food motives in participants [32]. This questionnaire has 2 to 4 questions for each of the nine motivations mentioned in the introduction section, examined both before and during COVID-19. The score range for answering the questions is a 4-point scale (very important = 4, relatively important = 3, of little importance = 2, and not important = 1). For the importance of each factor or motivation of food choice, the score of the related questions was averaged to determine its importance compared to other factors or motivations. Considering the questions before and during COVID-19, the changes in the importance of motivation in food choices were also examined.

### 2.5 Statistical analysis

The data were analyzed using the R software 4.1.2 version. Depending on the data distribution, data were presented as central and dispersion indices such as mean, median, standard deviation, and mid-quarter amplitude. Linear regression was used to determine the effect of independent variables, but Analysis of variance was used to determine differences between groups. The significance level in this study was considered five percent (*p* = 0.05).

## 3 Results

The number of participants who completed the questionnaire was 366. By excluding some questionnaires due to lacking required information or the person being out of the age range, 331 people entered the study data analysis. Based on the collected data, the findings were analyzed into relevant tables. First, the distribution frequency of participants’ demographic variables and, later, the mean of quantitative variables were compared. The mean age of respondents in the present study was 39.65±13.13 years, and 69.2% of the participants in the study were of male gender. Most participants did not report any change in their employment status (65.3%) or income status (58.0%). The rest of the respondents’ demographic data are shown in Table 1.

**Table 1:** Demographics of the final respondents.

**Mean age ± SD**	39.65 ± 13.13
	**N = 331 (100%)**
**Sex**	
Male	229 (69.2)
Female	102 (30.8)
**Marital status**	
Not currently married	90 (27.2)
Currently married	241 (72.8)
**Housing status**	
Owner	184 (55.6)
Rental	68 (20.5)
Living with parents	79 (23.9)
**Education level**	
Under-diploma	32 (9.7)
Diploma degree	53 (16.0)
Bachelor degree	161 (48.6)
Master or higher degree	85 (25.7)
**Employment status**	
Unemployed / Housewife	53 (16.0)
Retired	75 (22.7)
Employee	87 (26.3)
Self employed	69 (20.8)
University student	47 (14.2)
**Change in employment status**	
Has not changed	216 (65.3)
Presence working hours were reduced	70 (21.1)
I became teleworking	26 (7.9)
I lost my job	19 (5.7)
**Change in income**	
Has not changed	192 (58.0)
Has decreased	109 (32.9)
Has increased	30 (9.1)
**COVID-19 positive test history for the individual**	
Yes	135 (40.8)
No	196 (59.2)
**COVID-19 positive test history for the relatives**	
Yes	270 (81.6)
No	61 (18.4)

### 3.1 Fear of COVID-19

The results showed that the mean score of fear of COVID-19 in the study population was 15.25± 5.78. Women showed significantly higher levels of fear than men (*p* < 0.001). The findings in Table 2 indicate the mean fear caused by COVID-19 in different subgroups of the respondents. Among the subgroups related to housing status, people living in owned houses showed significantly more fear than those living with parents (*p* = 0.025). Among the subgroups related to employment status, the unemployed/ housewives group showed significantly more fear than other employment status groups (*p* = 0.001). For the career change subgroup, those who lost their jobs and those who became teleworking showed significantly more fear than those with secure unchanged job status (*p* = 0.04).

**Table 2:** Comparison of the fear of COVID-19 in different subgroups of respondents.

Variable/Subgroup	Mean Fear of COVID-19	*p*-Value
**Sex**		
Male	14.07 ± 5.31	**<0.001**
Female	17.90 ± 5.92	
**Marital status**		
Not currently married	14.78 ± 6.18	0.382
Currently married	15.43 ± 5.62	
**Housing status**		
Owner	15.99 ± 5.32^**a**^	**0.025**
Rental	14.74 ± 6.79^ab^	
Living with parents	13.99 ± 5.64^**b**^	
**Education level**		
Under-diploma	14.97 ± 5.07^a^	0.732
Diploma degree	15.98 ± 5.78^a^	
Bachelor degree	15.27 ± 6.16^a^	
Master or higher degree	14.87 ± 5.32^a^	
**Employment status**		
Unemployed / Housewife	18.23 ± 4.99^**a**^	**0.001**
Retired	14.32 ± 5.12^**b**^	
Employee	15.43 ± 6.18^**b**^	
Self employed	14.42 ± 5.61^**b**^	
University student	14.30 ± 6.11^**b**^	
**Change in employment status**		
Has not changed	14.82 ± 5.43^**a**^	**0.040**
Presence working hours were reduced	15.11 ± 6.03^ab^	
I became tele-working	17.50 ± 7.30^**b**^	
I lost my job	17.58 ± 5.57^**b**^	
**Change in income**		
Has not changed	15.05 ± 5.57^a^	0.181
Has decreased	15.97 ± 6.00^a^	
Has increased	13.97 ± 6.08^a^	
**COVID-19 positive test history for the individual**		
Yes	15.35 ± 5.08	0.798
No	15.19 ± 6.22	
**COVID-19 positive test history for the relatives**		
Yes	15.35 ± 5.70	0.552
No	14.84 ± 6.13	

Note: For each variable, **non-common letters** indicate a **significant difference** at the level of 5% **(*p*** **<0.05)**

### 3.2 Food choice motives

Findings from the study population data analysis showed that the most important food choice motivations were related to sensory appeal and health in both pre-and post-epidemic periods. On the contrary, the least important food choice motives were Ethical concern and Convenience, respectively. The results for the mean of each motivation before and during the COVID-19 epidemic are listed in Table 3. The only change in the ranking of motivations is associated with shifting Prices and Mood motivations to the third and fourth placements. Before COVID-19, Price was more important than Mood, but Mood became more important during the epidemic.

**Table 3:** Comparison of food choice motives between before and during the COVID-19.

Food Choice Motives		Mean ± Standard deviation		*p*-Value
		Before of COVID-19	During of COVID-19	
1.	Sensory appeal	3.32 ± 0.73	3.31 ± 0.69	0.877
2.	Health	3.20 ± 0.79	3.26 ± 0.72	**0.020**
3.	Price	3.06 ± 0.87	3.03 ± 0.80	0.288
4.	Mood	3.03 ± 0.90	3.09 ± 0.79	0.144
5.	Natural content	2.98 ± 0.92	2.99 ± 0.80	0.680
6.	Familiarity	2.96 ± 0.87	2.96 ± 0.80	0.966
7.	Weight control	2.87 ± 0.87	2.86 ± 0.81	0.628
8.	Convenience	2.81 ± 0.84	2.83 ± 0.76	0.584
9.	Ethical concern	2.60 ± 0.97	2.56 ± 0.97	0.126

A comparison of food choice motives showed that only the mean of Health during COVID-19 increased significantly compared to the before state (*p* < 0.020). The mean of Mood, Convenience, and Natural content also increased during the epidemic, but the differences were insignificant. In contrast, the mean of Price, Weight control, Sensory appeal, Familiarity, and Ethical concern decreased, which were also insignificant.

### 3.3 Fear of COVID-19 and food choice motives

The findings in Table 4 indicate the relationship between fear of COVID-19 and food choice motives. Food motivations Sensory appeal, Health, and Weight control were insignificant and showed no relationship. On the contrary, other motives were positively associated with the fear of COVID-19. Mood and Familiarity indicated a positively significant relationship. Nevertheless, Price, Natural content, Convenience, and Ethical concerns were motivations whose positive association with fear of COVID-19 was small but significant (*p*<0.05).

**Table 4:** The relationship between fear of COVID-19 and food choice motives.

Food Choice Motives		Fear of COVID-19	*p*-Value
		Beta (95% CI)	
1.	Sensory appeal	0 (-0.01, 0.01)	0.415
2.	Health	0 (0, 0.01)	0.241
3.	Price	0.01 (0, 0.02)	**0.012**
4.	Mood	0.02 (0.01, 0.03)	**< 0.001**
5.	Natural content	0.01 (0, 0.02)	**0.032**
6.	Familiarity	0.02 (0.01, 0.03)	**0.002**
7.	Weight control	0.01 (0, 0.02)	0.161
8.	Convenience	0.01 (0, 0.02)	**0.017**
9.	Ethical concern	0.01 (0, 0.02)	**0.031**

## 4 Discussion

This study aimed to determine the relationship between fear of COVID-19 and food choice motives in people living in Qazvin. This study is the first to investigate the relationship between fear of COVID-19 and food choices in Iran. COVID-19 can affect people’s food choices during the epidemic by changing the environment and circumstances. Therefore, the result of the present study can be helpful to education and nutritional interventions to combat this epidemic and other future infectious diseases.

### 4.1 Discussion about the mean fear of COVID-19 and its comparison in demographic subgroups

In the present study, the respondents’ mean fear of COVID-19 was 15.25±5.78. In the study of Zamanian *et al*., conducted at the advent of COVID-19 in Iran in March 2020, the mean level of fear of COVID-19 in six cities of Iran was 15.68±0.46. The critical point about this study is that the questionnaire used to measure the fear of COVID-19 consisted of five questions with a score range of 4 to 20 [33]. Compared to the questionnaire used in the present study with a range of 7 to 35 (7 questions), although the mean fear of COVID-19 was approximately the same in the two studies, the number related to the study of Zamanian *et al*. showed more fear of COVID-19. On the other hand, the timing of their study related to the days when the disease had just arrived in the country, and not much information was present about it. This lack of information, along with the lack of vaccination, treatment, and practical strategies against the disease and the high mortality rate of COVID-19 in different parts of the world, caused more fear in the community.

In the study of Akbarpour *et al*., the fear of COVID-19 was measured for 1223 Iranians via an online questionnaire posted on social networks. The questionnaire used for this study was the same 7-item questionnaire as the present study and showed the mean fear of COVID-19 equal to 19.70±5.08. Their study timing, related to the summer of 2020, can effectively show a higher fear score of COVID-19. Besides, about two-thirds (70%) of the participants in the study were female. The significant difference in the level of COVID-19 fear in females compared to males in this study (*p*<0.05) is another reason why the mean fear of COVID-19 in the Akbarpour *et al*. study is high [34].

The demographic data analysis in the current study showed that among the subgroups related to housing status, people who live in owned houses showed significantly more fear than those living in their parents’ (*p* = 0.025). This can be because of marriage, more living costs, and other related concerns. Contrariwise, for those living with their parents, being younger, single, and spending lower costs can cause less fear of COVID-19. Among the subgroups related to employment status, unemployed people and housewives showed significantly more fear than other job statuses (*p*<0.05). The housewife/unemployed subgroup may be more afraid of the disease because they stay longer at home and are more influenced by the media and COVID-19 news. In contrast, those physically present and busy at work are less likely to follow COVID-19 news, and due to knowing they are exposed to the virus, they get used to the idea of its presence. Those who lost their jobs or shifted to working from home also reported more fear of COVID-19 than the unemployed/housewives group due to the loss of income and other psychological effects of this event. Further research can confirm or disprove these assumptions.

In the present study, women showed significantly higher levels of fear than men (*p*<0.001). Many studies revealed strong linkages between gender and COVID-19-related fear and anxiety, suggesting that females perceive coronavirus as a greater threat to personal health and population than males, suggesting a greater psychological vulnerability in women during the COVID-19 pandemic.

Metin *et al*. (2022) meta-analysis showed that the female gender factor has a statistically significant effect on COVID-19-related fear (ES = 0.307; 95% CI = [0.255,0.359]) and anxiety (ES = 0.316; 95% CI = [0.183,0.449]) [35]. Another study by Broche-Pérez *et al*. (2022) in Cuba showcased that females with middle and high fear levels (compared to low fear) experienced 3.13 and 3.45 times more fear than males, respectively [36]. A similar correlation between gender and fear of COVID-19 was found in the studies by Akbarpour *et al*. (2022), Reznik *et al*. (2020), and Bitan *et al*. (2020) and the current study [14, 34, 37].

In the study by Zamanian *et al*. (2020), both men and women exhibited similar levels of fear towards COVID-19, contrasting with the current study and other previously cited studies. The correlation between education level and fear of COVID-19, as discovered by Zamanian *et al*. (2020), is significant. They declared that fear of COVID-19 increased with an increase in education level [33]. However, the current study reveals no significant relationship between education level and fear of COVID-19.

The results of the present study found that fear of COVID-19 has decreased compared to similar studies, with a lower mean level of fear observed. There is also a gender-related difference in the fear of COVID-19, with women showing higher concern towards this disease.

### 4.2 Discussion about the mean score of food choice motives before and during the COVID-19 and their comparison between the two periods

The analysis in the present study showed that both before and during the epidemic, the most important food choice motivations were related to sensory appeal and health, respectively. On the contrary, the least important food choice motives were related to Ethical concern and Convenience, respectively. The only change in the ranking of motivations before and during the epidemic was the shift in Price and Mood motivations to the third and fourth positions. Since the onset of the COVID-19 pandemic, Mood has become a significant factor, surpassing Price in importance, which was previously considered more critical. This change aligns with findings from several studies [1, 38].

Romeo-Arroyo *et al*. (2020) measured food choice motivations in 600 Spanish people in the COVID-19 quarantine. Their study findings showed that Sensory appeal, Health, and Mood are the most important factors, respectively. The results of this study were on par with the present study regarding the importance of the mentioned motives during COVID-19 [38].

A recent study by Sorić *et al*. (2021) examined the changes in food choice motives in Croatia during the COVID-19 pandemic, compared to pre-pandemic. The study found that the top three motivations for food choices before the pandemic were Sensory appeal, Health, and Convenience, respectively. During the epidemic, the same three factors were ranked first to third: Health, Convenience, and Sensory appeal. Ethical concerns were also the least important among food motivations in both periods. The results of the present study align with the findings of the study by Sorić *et al*. (2021), highlighting the significance of Sensory appeal and Health as the most important motivators. However, in their results, Convenience was in third place (before the pandemic) and second place (during the epidemic), discordant with our results of the Convenience factor being in eighth place in both periods. However, the present study aligned with their results, placing Ethical concerns at the bottom [39].

In the present study and the studies by Snuggs *et al*. (2021) and Sorić *et al*. (2021), Sensory appeal and Health were the most important factors before and during the epidemic [39, 40]. In the study by Marty *et al*. (2021) in France, Sensory appeal was considered the most important before and during COVID-19, which is in line with our results. Nevertheless, their findings displayed Natural content in the second place [1].

Comparison of the change in each food choice motive in the present study showed that only the change in the mean Health during COVID-19 was significant compared to before COVID-19 and increased (*p* = 0.02). The mean of Mood, Convenience, and Natural content also increased, just like in some other studies during the epidemic [1, 39], but the mean differences were insignificant in the present study. In contrast, the mean of Price, Weight control, Sensory appeal, Familiarity, and Ethical concerns decreased were insignificant.

Sorić *et al*. (2021) revealed that the importance of motivations for Health, Convenience, Natural content, Price, Weight control, Familiarity, and Ethical concerns increased significantly during the COVID-19 quarantine period compared to the pre-epidemic. These results were consistent with the present study as both showed a boost in importance and significance in Health and an increase in the importance of Convenience and Natural content [39].

The findings of Marty *et al*. (2021) study proved that the changes in the importance of all food motivations during COVID-19 were significant compared to pre-pandemic, which indicates a decrease in the importance of Familiarity, Convenience, and Price. Their concurrent results with the present study showed a significant increase in the importance of Health. The upsurge in the importance of Health, Mood, and Natural content and the decline in the importance of Familiarity and Price were aligned with the changes in the present study. The change in the importance of other motives was inconsistent [1].

Snuggs *et al*. (2021) showed the change in the importance of Familiarity was significantly decreasing, and for Weight control, Health and Mood were significantly increasing. The change in the importance of Health was consistent with the present study [40].

The reason behind having more changes in food choice motives or a greater number of motives that differed significantly between pre- and during the COVID-19 periods may have occurred due to differences in the timing of the current study and the other relative studies. Differences in the demographic characteristics of the studies may also explain differences in food choice motives of the compared studies. Many studies were conducted during a short quarantine period or post-COVID-19; however, the present study investigated the long-term impact of COVID-19 in the community. This stable situation in which the country has reached resilience, and people are more familiar with this virus. In almost all studies analyzing changes in food choice motives, over 70% of the study population was female. Findings from the present study and other studies showed that the change in the importance of food motivation was more remarkable for females [39, 41]. Consequently, females may contribute to more substantial changes in motivation, given their higher representation. Notably, in our current study, the gender distribution differed from the previous studies, with 70% of the population being male. This stands in contrast to similar studies where a predominantly female participant base often resulted in less apparent changes in motivation among males [1, 41].

### 4.3 Discussion about the relationship between fear of COVID-19 and food choice motives

The present study’s findings showed that all food motives except Sensory appeal, Health, and Weight control were positively associated with fear of COVID-19. This positive correlation demonstrates that COVID-19 fear made people pay more attention to food choices. Price, Mood, Natural content, Familiarity, Convenience, and Ethical concern were the motivations positively and significantly related to fear of COVID-19 (*p*<0.05). As the fear of COVID-19 grows, the importance of the mentioned factors or motivations increases regarding food choice or preparation. Generally, people who are more afraid of the virus cautiously prefer consuming more foods with no artificial additives, as such a choice makes them feel secure.

Shen *et al*. (2020) explored the relationship between stress and motivations of Mood, Convenience, Price, Familiarity, and Natural content [42]. Equating fear with stress, the same motivations related to the fear of COVID-19 in the present study. As a result, the mentioned food choice motives may relate to COVID-19 psychological factors such as fear, stress, and anxiety. The current study is the first to investigate how fear of COVID-19 affects Iranian people’s food choices.

### 4.4 Limitations of the study

While this study provides valuable insights into the relationship between fear of COVID-19 and food choice motives, several potential limitations can be acknowledged. Firstly, the study’s cross-sectional design limits our ability to establish causality between fear of COVID-19 and food choice motives. Longitudinal studies would provide a more comprehensive understanding of how these variables interact over time. Additionally, the study relied on self-reported data, and despite the research team trying their best to minimize any type of bias throughout the entire process, but chances are recall bias and social desirability bias can still exist. Participants may have provided responses that they perceived as socially acceptable or aligned with their personal beliefs and mentality, potentially affecting the validity of the findings. Also, the study was conducted in a specific geographic area (Qazvin, Iran), which may limit the generalizability of the results to other populations from other regions with different socio-cultural contexts. Lastly, while efforts were made to achieve a representative sample, sampling bias may have influenced the findings, particularly considering the reliance on online surveys. Despite these limitations, this study provides valuable insights into the psychological factors influencing food choices and preferences during the COVID-19 pandemic for future research in this area.

## 5 Conclusion

Many psychological factors, including fear of COVID-19, influence food choice motives. Such correlations indicate that people have become more conscious of their food choices due to the COVID-19 pandemic. Health and Natural content have increasingly gained importance among food choices, as experts advise on the benefits of eating healthy to combat COVID-19. These guidances are proving to impact people’s food choices positively. Sensory appeal remains the top-ranking priority for most individuals before and during the pandemic, highlighting the significance of nutrition sensory effects in specific conditions like the pandemic. Food motivations such as Mood, Familiarity, Price, Natural content, Convenience, and Ethical concerns were positively linked to the fear of COVID-19. Further research on the relationship between fear of COVID-19 and food choice motives will provide better insights for future comparison and generalization of results.
