# Correction: The relationship between fear of COVID-19 and food choice motives in the Iranian population

**DOI:** 10.1371/journal.pone.0317464

**Published:** 2025-01-07

**Authors:** Seyyed-Ali Hoseinean, Bita Rahmani, Ahad Alizadeh, Maryam Javadi, Roghieh Nooripour, Atieh Razzazi, Mohammad Reza Shiri-Shahsavar

An additional affiliation is missing for the fifth author. Roghieh Nooripour is also affiliated with the Department of Counseling, Qazvin Branch, Islamic Azad University, Qazvin, Iran.
